# Clinical and laboratory predictors of mortality in *Staphylococcus aureus* bacteremia in a high-risk setting: a single-center retrospective analysis of Pitt score, SOFA, neutrophil-to-lymphocyte ratio, and platelet-to-lymphocyte ratio

**DOI:** 10.1080/07853890.2025.2573984

**Published:** 2025-10-21

**Authors:** Yeliz Özdemir, İrem Aşkın Yılmaz, Süheyla Serin Senger, Şebnem Çalık

**Affiliations:** a Department of Infectious Diseases and Clinical Microbiology, Ministry of Health Izmir City Hospital, Izmir, Turkey; b Department of Infectious Diseases and Clinical Microbiology, Izmir Faculty of Medicine, University of Health Sciences Turkey, Izmir City Hospital, Turkey

**Keywords:** *Staphylococcus aureus*, bacteremia, neutrophil-to-lymphocyte ratio, Pitt bacteremia score, mortality

## Abstract

**Objective:**

*Staphylococcus aureus* bacteremia (SAB) is associated with significant morbidity and mortality despite advancements in antimicrobial therapies. Timely risk stratification is critical to optimize patient outcomes. This study aimed to evaluate the prognostic value of clinical (Pitt bacteremia score [PBS]), combined clinical and laboratory (Sequential Organ Failure Assessment [SOFA]), and laboratory-based parameters (neutrophil-to-lymphocyte ratio [NLR] and platelet-to-lymphocyte ratio [PLR]) in patients with SAB.

**Methods:**

We conducted a retrospective observational study of 150 adult patients diagnosed with monomicrobial SAB between November 2023 and November 2024 at a tertiary care center. The predictive value of PBS, SOFA, NLR, and PLR for in-hospital mortality was assessed using ROC analysis and logistic regression.

**Results:**

The overall in-hospital mortality rate was 46%. PBS >4 and NLR >21 were independently associated with increased mortality (OR 11.72 and 6.36, respectively; *p* < 0.001). Absence of fever (OR 0.11) and presence of central venous catheter (OR 4.11) were also significant predictors. ROC analysis demonstrated good predictive performance for both PBS (AUC: 0.82) and SOFA (AUC: 0.795) scores with no statistically significant difference between them. Community-acquired SAB and primary bacteremia were associated with worse prognosis. Methicillin resistance did not significantly affect mortality. Fever response was significantly blunted in older adults but not in immunosuppressed patients.

**Conclusion:**

High PBS and NLR are strong and independent predictors of mortality in SAB. As this study was conducted in a high-risk cohort with a considerable proportion of critically ill and immunocompromised patients, the findings are particularly relevant to this vulnerable population. External validation in larger, multicenter cohorts is required to confirm their generalizability.

## Introduction

*Staphylococcus aureus* is a Gram-positive pathogen responsible for a wide range of serious and potentially life-threatening infections. Its systemic dissemination is facilitated by various virulence factors, immune evasion strategies, and its ability to form biofilms [1]. Among the most critical clinical manifestations caused by *S. aureus* is bacteremia, which is associated with high morbidity and mortality rates. The mortality rate of *S. aureus* bacteremia (SAB) ranges between 10% and 30%; although a relative decline has been observed over the past three decades, approximately one in four patients with SAB still die within 90 days of diagnosis [2]. Despite the availability of effective antimicrobial agents, SAB continues to pose a significant mortality risk, underscoring the need for continued research and improvement in clinical management.

The Pitt Bacteremia Score (PBS) and the Sequential Organ Failure Assessment (SOFA) score are widely utilized tools for assessing disease severity in patients with SAB. PBS evaluates solely clinical parameters, assigning scores from 0 to 14, with scores ≥4 generally indicating critical illness and a higher risk of mortality [3]. In contrast, the SOFA score (SS) incorporates both clinical and laboratory parameters to assess organ dysfunction. Three of the SS’s six components are laboratory-based, highlighting the essential role of biochemical and hematological tests in clinical decision-making. The neutrophil-to-lymphocyte ratio (NLR) and the platelet-to-lymphocyte ratio (PLR) are hematological markers that reflect systemic inflammation and immune response. Upon exposure to physiological stressors, the immune system initiates a compensatory response marked by neutrophilia, thrombocytosis, and concurrent lymphopenia. Consequently, elevated NLR and PLR values have been identified as potential prognostic indicators of adverse outcomes in patients with bacteremia [4,5].

In recent years, the incidence and complexity of SAB have been increasing, largely due to the rise in invasive medical procedures and the expanding population of immunocompromised individuals [6–8]. This increasing incidence imposes a considerable clinical burden on healthcare systems. Although current prognostic scoring systems—particularly the PBS and the SS—are frequently employed to predict sepsis outcomes, they present notable limitations. While PBS is based primarily on early clinical observations, scoring systems such as the SS rely on biochemical markers of organ dysfunction. However, the time required to obtain these laboratory parameters can delay diagnosis and therapeutic interventions. Moreover, the diagnostic sensitivity of these scoring systems remains limited—particularly in immunocompromised patients—due to the atypical and often subtle presentation of infections in this group. In this study, we aimed to retrospectively evaluate the role and prognostic value of clinical and laboratory indicators—particularly inflammatory markers such as the PBS, SS, NLR, and PLR—as independent risk factors for mortality in SAB, based on a cohort of 150 cases. The findings of this study are expected to contribute valuable evidence to support clinical decision-making and optimize the management strategies for these high-risk infections.

## Materials and methods

This single-center, retrospective observational study was conducted at a tertiary education and research hospital. It included patients over 18 years of age who were hospitalized and followed up during the period in which *S. aureus* was isolated from at least one blood culture. Patient records dated from 01 November 2023 to 01 November 2024 were retrospectively accessed through the hospital’s electronic database for research purposes. All consecutive cases of SAB were identified from the hospital microbiology database, which captures every positive blood culture during the study period, thereby minimizing selection bias. To evaluate the specific impact of pure *SAB* on mortality, patients with polymicrobial infections—defined as the presence of other microorganisms isolated from blood, urine, sputum, or other culture samples—were excluded from the study. Only clinically significant microorganisms were included in the analysis, and potential contaminants such as coagulase-negative staphylococci were excluded in accordance with CDC/NHSN criteria [9]. Patients with blood cultures yielding an additional isolate considered a true pathogen (e.g. *Enterococcus spp.*, *Pseudomonas spp.*, or *Candida spp.*) were excluded from the study. Accordingly, the definition of polymicrobial bacteraemia in this study refers specifically to these excluded cases. All patient data were retrospectively retrieved from the hospital’s electronic medical record system. Demographic characteristics, methicillin resistance status of the isolates, clinical and laboratory features of the infections, administered treatments, and treatment outcomes were systematically recorded. The severity of illness was evaluated using the PBS and the SOFA score. The PBS (range 0–14) incorporates parameters such as body temperature, blood pressure, mechanical ventilation requirement, mental status, and recent cardiac arrest, with higher scores indicating more severe disease [10]. The SOFA score was calculated to assess dysfunction across six organ systems (respiratory, coagulation, liver, cardiovascular, central nervous system, renal), each rated from 0 to 4 points, with the total score reflecting the degree of organ failure [11]. All variables required for the calculation of these scores were extracted at the time of the first positive blood culture. Patients with missing data for these variables were excluded from the study.

Episodes of SAB were classified according to Friedman et al. [12] as healthcare-associated (HCA-SAB), hospital-acquired (HA-SAB), or community-acquired (CA-SAB). HA-SAB was defined as onset ≥48 h after admission. HCA-SAB was defined as community-onset SAB with prior healthcare exposure (hospitalization, dialysis, intravenous therapy, long-term care facility). CA-SAB was defined as community-onset SAB without such exposures.

Clinical manifestation of infection was defined as the infection presentation associated with SAB at the time of diagnosis. Categories included bacteremia, pneumonia, urinary tract infection, skin and soft tissue infection, central venous catheter-related infection, and endocarditis.

Fever was defined as a body temperature ≥38.0 °C at the time of SAB diagnosis.

Presence of a catheter was defined as the presence of a central venous catheter (CVC) at the time of SAB diagnosis.

Mortality was defined as all-cause in-hospital mortality, i.e. death from any cause occurring during the index hospitalization. In addition, 23 comorbidities and potential risk factors were recorded and analyzed for their association with mortality using Pearson’s Chi-square and Fisher’s exact test (*p* < 0.05 considered statistically significant).

## Statistical analysis

Data were analyzed using SPSS version 26 and MedCalc version 19. The normality of continuous variables was assessed through graphical methods, normality tests, and consideration of sample size. For variables that did not conform to a normal distribution, comparisons between independent groups were conducted using the Mann-Whitney U test. Results were expressed as median values along with interquartile ranges (IQR, 25th–75th percentile).

Receiver Operating Characteristic (ROC) analysis was employed to evaluate the diagnostic performance of continuous variables, and cutoff values were determined using the Youden Index. Based on these cutoffs, dichotomous variables were created.

Categorical variables were summarized as frequencies and percentages using cross-tabulations. Their distributions were compared using Chi-Square test methods, and univariate odds ratios (ORs) were calculated. Due to insufficient sample sizes in certain diagnostic subgroups, distributional comparisons between survivors and non-survivors were not feasible and were thus marked as ‘not applicable (na)’.

ROC curve analysis was also applied to compare the predictive performance of the PBS and the SS with respect to mortality. The area under the curve (AUC) for each was calculated, and the statistical significance of the difference between them was assessed using the DeLong test.

Independent variables that demonstrated statistically significant associations in univariate analyses were included in a multivariate logistic regression model. The backward stepwise (Wald) method was used to determine the final model, and multivariate ORs were reported.

A two-tailed *p*-value < 0.05 was considered statistically significant for all analyses, and the type I error rate (α) was set at 0.05.

## Results

A total of 150 patients with *S.aureus* isolated from blood cultures were included in the study. The median age of the cohort was 66 years (interquartile range [IQR]: 55–74). Of the patients, 55% were male (*n* = 83). Comorbid conditions were present in 88% of the cases, and 52% of these patients were classified as immunocompromised (Supplementary Tables 1 and 2).

**Table 1.** Evaluation of the association between quantitative variables and in-hospital mortality.

	Total (*n* = 150)	Non-survivors (*n* = 69)	Survivors (*n* = 81)	
Variable	Median (IQR)	Median (IQR)	Median (IQR)	*p* value
Age (years)	66 (55–74)	68 (63–78)	61 (52–69)	<0.001
Time from admission to culture (days)	1.5 (0–7)	1 (0–9)	2 (0–6)	0.423
Duration of bacteremia (days)	4 (2–7)	4 (2–6)	5 (3–8)	0.115
PBS	3 (1–6)	5 (3–8)	2 (1–3)	<0.001
SOFA score	6 (2–10)	10 (6–13)	4 (1–7)	<0.001
White blood cell count (cells/µL)	11,950 (8,050–17,670)	13,310 (9,100–19,560)	10,880 (7,710–16,070)	0.020
Neutrophils (cells/µL)	10,105 (6,430–15,510)	12,060 (7,320–18,290)	9,250 (5,630–12,930)	0.009
Lymphocytes (cells/µL)	905 (490–1,280)	790 (360–1410)	930 (600–1,250)	0.249
Monocytes (cells/µL)	685 (390–960)	530 (280–870)	750 (460–1,040)	0.028
Platelets (cells/µL)	199,500 (127,000–276,000)	224,000 (143,000–300,000)	193,000 (116,000–252,000)	0.080
CRP (mg/L)	179 (97–256)	199 (128–263)	144 (64–237)	0.031
PCT (ng/mL)	2.41 (0.62–18)	6.4 (1.13–25)	1.32 (0.42–9.1)	0.012
NLR	11 (6–24.1)	17.1 (8.4–29.6)	9.5 (5.6–14.9)	0.002
PLR	232 (133–350)	283 (159–470)	199 (130–290)	0.006

CRP: C-reactive protein; NLR: Neutrophil-to-lymphocyte ratio; PBS: Pitt bacteremia score; PCT: Procalcitonin; PLR: Platelet-to-lymphocyte ratio; SOFA: Sequential organ failure assessment; IQR: Interquartile Range.

*P*-values were calculated using the Mann–Whitney *U*-test.

Regarding risk factors for methicillin-resistant *S. aureus* (MRSA), 57% of patients had a history of hospitalization within the previous three months, and 66% had been hospitalized within the past year. In terms of infection origin, 86 (57.3%) cases were classified as healthcare-associated SAB, 38 (25.3%) as hospital-acquired SAB, and 26 (17.3%) as community-acquired SAB according to the Friedman classification (Supplementary Table 3).

When the distribution of infection sources was analyzed, the most frequently identified diagnoses were primary bacteremia (32%) and pneumonia (31%), followed by skin and soft tissue infections (15%), urinary tract infections (11%), and catheter-related infections (7%). Transthoracic echocardiography (TTE) was performed in 60% of patients, while transesophageal echocardiography (TEE) was conducted in only 1%. The incidence of infective endocarditis was found to be 1%. The prevalence of MRSA among the isolates was 30% (Supplementary Table 4). However, methicillin resistance did not show a statistically significant association with mortality (*p* = 0.174). Overall, all-cause in-hospital mortality occurred in 69 of 150 patients (46%). In additional analyses, none of the comorbidities or risk factors evaluated showed a statistically significant independent association with mortality.

When the association between patients’ quantitative variables and mortality was examined, the levels of age, PBS, SS, white blood cell count, neutrophil count, procalcitonin, C-reactive protein (CRP), NLR, and PLR were found to be significantly higher in deceased patients. In contrast, the monocyte count was significantly lower among those who died (*p* < 0.05) (Table 1).

The results of the ROC analysis for the patients’ quantitative variables are presented in Table 2. Based on the Youden index, the optimal cutoff values for predicting mortality were determined as follows: >4 for the PBS (sensitivity 81%, specificity 73%), white blood cell count >12030/cells/µL (sensitivity 61%, specificity 62%), neutrophil count >12930/cells/µL (sensitivity 48%, specificity 75%), lymphocyte count ≤480/cells/µL (sensitivity 39%, specificity 88%), monocyte count ≤600/cells/µL (sensitivity 54%, specificity 67%), CRP >116 mg/dL (sensitivity 78%, specificity 41%), procalcitonin (PCT) >5. 73 μg/L (sensitivity 54%, specificity 73%), NLR >21 (sensitivity 86%, specificity 37%), PLR >262 (sensitivity 57%, specificity 70%).

**Table 2.** ROC analysis of biomarkers and their optimal cut-off values for predicting mortality in *Staphylococcus aureus* bacteremia.

Variable	AUC (95% CI)	*p* (Area = 0.5)	Youden Index *J*	Cut-Off Value	Sensitivity (%)	Specificity (%)
PBS	0.820 (0.749–0.878)	<0.001	0.54	>4	81	73
White blood cell count	0.611 (0.528–0.689)	0.017	0.23	>12,030	61	62
Neutrophils	0.625 (0.542–0.702)	0.007	0.23	>12,930	48	75
Lymphocytes	0.555 (0.471–0.636)	0.274	0.27	≤480	39	88
Monocytes	0.604 (0.521–0.683)	0.027	0.20	≤600	54	67
CRP	0.602 (0.519–0.681)	0.027	0.19	>116	78	41
PCT	0.635 (0.552–0.712)	0.003	0.26	>5.73	54	73
NLR	0.649 (0.567–0.726)	0.001	0.31	>21	45	86
PLR	0.632 (0.549–0.709)	0.005	0.27	>262	57	70

CRP: C-reactive protein; NLR: Neutrophil-to-lymphocyte ratio; PBS: Pitt bacteremia score; PCT: Procalcitonin; PLR: Platelet-to-lymphocyte ratio; ROC: Receiver operating characteristic; AUC: Area under the curve.

When risk factors for mortality were evaluated, the following factors were found to be statistically significant in the univariate analysis: age ≥65 years (*p* = 0.022), community-acquired infection (*p* = 0.020), presence of central venous catheter (*p* = 0.001), absence of fever (*p* < 0.001), PBS >4 (*p* < 0.001), SS ≥2 (*p* = 0.002), white blood cell count >12030/cells/µL (*p* = 0.006), neutrophil count >12930/cells/µL (*p* = 0.004), lymphocyte count ≤480/cells/µL (*p* < 0.001), monocyte count ≤600/cells/µL (*p* = 0.013), CRP >116 mg/dl (*p* = 0.014), PCT >5.73 μg/L (*p* = 0.002), NLR >21 (*p* < 0.001), PLR >262 (*p* = 0.001) (Table 3).

**Table 3.** Univariate analysis of risk factors associated with mortality.

Variable	Overall (*n*, %)	Non-survivors (*n*, %)	Survivors (*n*, %)	*p* ^a^	OR (95% CI)	*p* ^b^
Age						
≥65	76 (51%)	42 (61%)	34 (42%)	0.021	2.15 (1.12–4.14)	0.022
<65	74 (49%)	27 (39%)	47 (58%)	—	1 (reference)	—
Sex						
Male	83 (55%)	37 (54%)	46 (57%)	0.697	0.88 (0.46–1.68)	0.697
Female	67 (45%)	32 (46%)	35 (43%)	—	1 (reference)	—
Comorbidity						
Present	132 (88%)	59 (86%)	73 (90%)	0.539	0.65 (0.24–1.74)	0.388
Absent	18 (12%)	10 (14%)	8 (10%)	—	1 (reference)	—
Immunosuppression						
Present	78 (52%)	33 (48%)	45 (56%)	0.345	0.73 (0.39–1.4)	0.345
Absent	72 (48%)	36 (52%)	36 (44%)	—	1 (reference)	—
Hospitalization within last 3 months						
Yes	86 (57%)	40 (58%)	46 (57%)	0.884	1.05 (0.55–2.01)	0.884
No	64 (43%)	29 (42%)	35 (43%)	—	1 (reference)	—
Hospitalization within last year						
Yes	99 (66%)	47 (68%)	52 (64%)	0.74	1.19 (0.60–2.35)	0.614
No	51 (34%)	22 (32%)	29 (36%)	—	1 (reference)	—
Clinical manifestation of infection				—	—	—
Primary bacteremia	48 (32%)	18 (26%)	30 (37%)			
Pneumonia	46 (31%)	31 (45%)	15 (19%)			
Skin and soft tissue infection	23 (15%)	9 (13%)	14 (17%)			
Urinary tract infection	17 (11%)	9 (13%)	8 (10%)			
Catheter infection	11 (7%)	1 (1%)	10 (12%)			
Thrombophlebitis	3 (2%)	0 (0%)	3 (4%)			
Endocarditis	2 (1%)	1 (1%)	1 (1%)			
Epidemiologic classification of infection				0.030	0.35 (0.15–0.85)	0.020
Community-acquired	26 (17.3%)	17 (24.6%)	9 (11.4%)			
Hospital-acquired	38 (25.3%)	12 (17.4%)	25 (31.6%)			
Healthcare-associated	86 (57.3%)	40 (58%)	45 (57%)			
Heart Valve Prosthesis				0.129	0.35 (0.11–1.15)	0.085
Present	16 (11%)	4 (6%)	12 (15%)			
Absent	134 (89%)	65 (94%)	69 (85%)			
Joint Prosthesis				0.289	0.37 (0.07–1.91)	0.237
Present	8 (5%)	2 (3%)	6 (7%)			
Absent	142 (95%)	67 (97%)	75 (93%)			
Central Venous Catheter Presence				0.001	4.49 (1.89–10.64)	0.001
Present	112 (75%)	61 (88%)	51 (63%)			
Absent	38 (25%)	8 (12%)	30 (37%)			
Endocarditis				—	—	—
Present	2 (1%)	1 (1%)	1 (1%)			
Absent	148 (99%)	68 (99%)	80 (99%)			
Methicillin Resistance				0.174	1.73 (0.86–3.5)	0.126
Present	45 (30%)	25 (36%)	20 (25%)			
Absent	105 (70%)	44 (64%)	61 (75%)			
Echocardiography				—	—	—
TTE	90 (60%)	32 (46%)	58 (72%)			
TEE	3(2%)	0 (0%)	3 (3%)			
Not performed	57 (38%)	37 (54%)	20 (25%)			
Fever (≥38.0 °C)				<0.001	0.13 (0.06–0.27)	<0.001
Present	75 (50%)	17 (25%)	58 (72%)			
Absent	75 (50%)	52 (75%)	23 (28%)			
PBS				<0.001	14.19 (5.91–34.07)	<0.001
>4	50 (33%)	42 (61%)	8 (10%)			
≤4	100 (67%)	27 (39%)	73 (90%)			
SOFA Score				0.001	6.06 (1.97–18.61)	0.002
≥2	124 (83%)	65 (94%)	59 (73%)			
<2	26 (17%)	4 (6%)	22 (27%)			
WBC >12030 (cells/µL)	73 (49%)	42 (61%)	31 (38%)	0.006	2.51 (1.3–4.85)	0.006
Neutrophils >12930 (cells/µL)	53 (35%)	33 (48%)	20 (25%)	0.005	2.80 (1.4–5.58)	0.004
Lymphocytes ≤480 (cells/µL)	37 (25%)	27 (39%)	10 (12%)	<0.001	4.56 (2.01–10.36)	<0.001
Monocytes ≤600 (cells/µL)	64 (43%)	37 (54%)	27 (33%)	0.012	2.31 (1.19–4.48)	0.013
CRP >116 (mg/L)	102 (68%)	54 (78%)	48 (59%)	0.021	2.48 (1.20–5.10)	0.014
PCT >5.73 (ng/mL)	58 (39%)	36 (52%)	22 (27%)	0.002	2.93 (1.48–5.78)	0.002
NLR >21	42 (28%)	31 (45%)	11 (14%)	<0.001	5.19 (2.35–11.47)	<0.001
PLR >262	63 (42%)	39 (57%)	24 (30%)	0.001	3.09 (1.57–6.06)	0.001
Platelet ≤100000 (cells/µL)	25 (17%)	11 (16%)	14 (17%)	0.826	0.91 (0.38–2.15)	0.826

CRP: C-reactive protein; DYE: Skin and soft tissue infection; UTI: Urinary tract infection; NLR: Neutrophil-to-lymphocyte ratio; PBS: Pitt bacteremia score; PCT: Procalcitonin; PLR: Platelet-to-lymphocyte ratio; TTE: Transthoracic echocardiography; TEE: Transesophageal echocardiography.

^a^ Chi-Square Test  .

^b^ Mantel-Haenszel Common Odds Ratio Estimate.

Mortality was higher in community-acquired infections, and these patients had higher SS. In our study, the fever response was statistically significantly lower in patients aged 65 years and older (*p* = 0.009). There was no statistically significant difference in the fever response between the immunosuppressed and non-immunosuppressed patients (*p* = 0.74). In supplementary analyses, neutrophil and lymphocyte counts were compared according to fever status. Median neutrophil counts tended to be higher in afebrile patients, but the difference did not reach statistical significance, while lymphocyte counts were similar between groups (Supplementary Table 5).

Variables with statistically significant ORs in the univariate analysis were included as independent variables in the multivariate logistic regression model. Among these, the presence of central venous catheter was associated with a 4.11 fold increased risk of mortality (*p* = 0.016; OR = 4.11; 95% CI: 1.3–12.99), PBS >4 was associated with a 11.72 -fold increased risk (*p* < 0.001; OR = 11.72; 95% CI 4.17–33), and NLR >21 was associated with a 6.36 -fold increased risk (*p* = 0.001; OR = 6.36; 95% CI 2.17–18.61). In contrast, the presence of fever was associated with an 89% reduction in mortality risk (*p* < 0.001; OR = 0.11; 95% CI: 0.04–0.29) (Table 4).

**Table 4.** Multivariate logistic regression analysis of risk factors associated with mortality.

Variable	Univariate OR (95% CI)	*p* ^a^	Multivariate OR (95% CI)	*p* ^b^
Age (>65 vs. ≤65)	2.15 (1.12–4.14)	0.022	—	ns
Epidemiologic classification of infection	0.35 (0.15–0.85)	0.020	—	ns
Central Venous Catheter (Present vs. Absent)	4.49 (1.89–10.64)	0.001	4.11 (1.30–12.99)	0.016
Fever (Present vs. Absent)	0.13 (0.06–0.27)	<0.001	0.11 (0.04–0.29)	<0.001
PBS (>4 vs. ≤4)	14.19 (5.91–34.07)	<0.001	11.72 (4.17–33.00)	<0.001
SOFA score (≥2 vs. <2)	6.06 (1.97–18.61)	0.002	—	ns
White blood cell count (>12030 vs. ≤12030)	2.51 (1.30–4.85)	0.006	—	ns
CRP (>116 vs. ≤116)	2.48 (1.20–5.10)	0.014	—	ns
PCT (>5.73 vs. ≤5.73)	2.93 (1.48–5.78)	0.002	—	ns
NLR (>21 vs. ≤21)	5.19 (2.35–11.47)	<0.001	6.36 (2.17–18.61)	0.001
PLR (>262 vs. ≤262)	3.09 (1.57–6.06)	0.001	—	ns

CRP: C-reactive protein; NLR: Neutrophil/lymphocyte ratio; PBS: Pitt bacteremia score; PCT: Procalcitonin; PLR: Platelet/lymphocyte ratio; SOFA: Sequential organ failure assessment; Constant = −1.51.

^a^ Mantel-Haenszel Common Odds Ratio Estimate.

^b^ Logistic Regression Method: Backward Stepwise (Wald), OR: ODDS Ratio; ns: not significant.

The study compared the prognostic performance of the PBS and SS. The area under the curve (AUC) for PBS was 0.820 (95% CI: 0.749–0.878), while the AUC for the SOFA score was 0.795 (95% CI: 0.722–0.857). Although the AUC difference favored PBS (ΔAUC = 0.024), the confidence interval for the difference (−0.021 to 0.071) included zero, and the result was not statistically significant (*p* = 0.2917, DeLong test).

**Figure 1.**
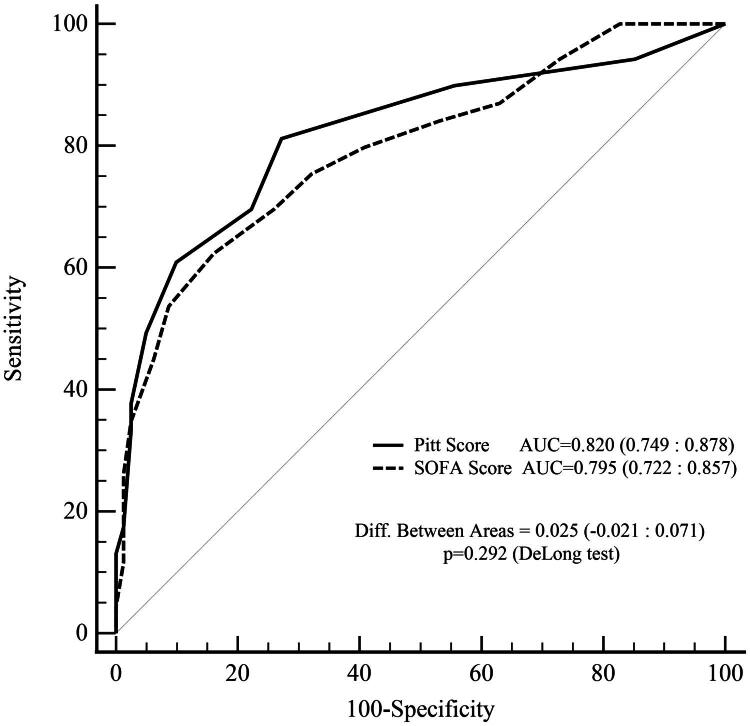
Receiver Operating Characteristic (ROC) curve comparison of the Pitt bacteremia score and SOFA score for predicting mortality in *Staphylococcus aureus* bacteremia.

Both scores were found to predict mortality with high accuracy (AUC > 0.75). Although the PBS exhibited a numerically higher AUC compared to the SOFA score, this difference was not statistically significant (*p* > 0.05). The inclusion of zero in the confidence interval for the difference further supports the comparable predictive performance of the two scoring systems (Figure 1).

## Discussion

SAB is a serious clinical condition that is frequently associated with significant complications and elevated mortality rates [13]. In cases of SAB, mortality rates as high as 30% have been reported [14]. Many studies on SAB have demonstrated that advanced age and primary bacteremia are independent predictors of mortality [15]. In our study, which predominantly comprised cases from these two groups, the mortality rate was determined to be 46%. The elevated mortality rate observed in our study, compared to previous reports, may be attributed to the inclusion of a high-risk patient population—namely, individuals of advanced age, those with multiple comorbidities, and immunosuppressed status. When assessing risk factors linked to mortality, absence of fever, presence of central venous catheter, and a PBS >4 emerged as significant clinical indicators, while among laboratory parameters, an elevated NLR was independently associated with increased mortality. Our findings highlight that a PBS exceeding 4 is a strong predictor of mortality. This finding further underscores the critical influence of systemic infection-related effects and concurrent organ dysfunction on mortality. Previous studies have demonstrated that a high PBS is associated with increased mortality in Gram-negative bacteremias and has shown superior predictive value compared to SIRS and quick SOFA scores [16]. In our study, the prognostic performance of PBS and SOFA scores in predicting mortality was compared, and it was shown that both scoring systems are equally effective. Similarly, the observation of a 6.36-fold increase in mortality risk among patients with NLR >21 highlights the critical role of systemic inflammatory severity in determining infection outcomes. Consistent with our findings, the literature supports that elevated NLR values reflect an exaggerated immune response to infection, which may contribute to organ dysfunction and increased risk of mortality [17,18]. High PBS and elevated NLR values, in particular, may serve as valuable tools for assessing infection severity and guiding early risk stratification to optimize treatment strategies. Furthermore, previous studies have demonstrated that the mortality rate associated with MRSA bacteremia is higher compared to MSSA bacteremia [15,19].

Over time, the standardization of SAB management protocols and the expanded availability of alternative antibiotics to vancomycin—effective against MRSA—have contributed to the convergence of mortality rates between MRSA and MSSA bacteremias [2]. Our study clearly showed that there was no statistically significant difference in mortality between MRSA and MSSA bacteremia. This result is consistent with recent literature, which reflects the impact of standardized SAB management protocols and improved access to alternative antimicrobial agents. Although methicillin resistance itself does not appear to influence clinical outcomes, factors such as the origin of infection, advanced age, and the presence of multiple comorbidities continue to significantly increase the risk of complications and mortality in SAB.

One of the key clinical characteristics that define complicated SAB is its community-acquired (CA) origin. CA SAB has been shown to increase the risk of complications by approximately threefold [20]. In hospitalized patients, clinical signs of fever and infection can be more readily identified through daily monitoring. In the absence of overt clinical findings, laboratory investigations can facilitate early diagnosis. Consequently, timely initiation of treatment and source control measures lead to shorter durations of bacteremia and fewer complications in nosocomial SAB compared to community-acquired SAB [2,21]. The prolonged time to diagnosis in cases of community-acquired SAB allowed for the development of vegetations, suggesting that community-acquired infections may also present a significant risk for the onset of endocarditis [21]. There are studies in the literature reporting mortality rates of up to 35%–40% in CA SAB [22,23]. In our study, CA SAB was identified as an independent variable associated with increased mortality, with a mortality rate of 66.6% observed in this group. Upon examining the factors contributing to this outcome, it was found that patients presenting with CA-SAB had a more severe clinical presentation, as reflected by higher SS in this subgroup.

Early diagnosis and timely treatment are essential for improving survival rates in infectious diseases. Fever, one of the cardinal signs of infection, is observed in approximately 73% of patients with SAB [24]. Fever represents one of the body’s natural defense mechanisms against infection and reflects an appropriately functioning immune response. In the study conducted by Willcox et al. all patients with SAB who failed to exhibit a febrile response succumbed to the infection, and the absence of fever was identified as a poor prognostic indicator [18]. A diminished febrile response to infection is frequently observed in elderly individuals and immunocompromised patients [25]. In our study, fever was present in 50% of the patients. The febrile response was significantly lower in patients aged 65 years and older, whereas no significant difference was observed between immunocompromised and non-immunocompromised individuals. The absence of a fever response was found to be a statistically significant factor associated with increased mortality. It is considered that the high proportion of geriatric patients—who tend to exhibit a diminished febrile response to infections—may have contributed to this finding.

Another critical factor influencing patient prognosis is the source of bacteremia. In studies evaluating SAB, primary bacteremia has consistently been reported as the most frequent clinical presentation [26,27]. Cases in which the source of infection remains unidentified and are classified as primary bacteremia are generally associated with higher mortality rates [28]. Endocarditis, a complication associated with high mortality, occurs in 5% to 64% of SAB cases initially classified as primary bacteremia and may be overlooked if echocardiography is not performed. Therefore, echocardiographic evaluation is recommended in cases of primary bacteremia that present a high risk of endocarditis [29]. Although current guidelines recommend performing echocardiography in all cases of SAB, real-world data indicate that the utilization rate of echocardiography ranges from 40% to 68% [30,31]. Primary bacteremia was the most frequently observed diagnosis in our study. Given that TTE was performed in only 60% of cases and TEE in merely 1%, it is plausible that some cases of endocarditis were overlooked and consequently misclassified as primary bacteremia. This potential underdiagnosis may have contributed to an overestimation of mortality rates within the primary bacteremia group. Inadequately treated cases with missed diagnoses are likely to re-emerge in clinical practice as relapses. Indeed, previous SAB cohorts report infective endocarditis rates of approximately 10–20%, highlighting that our observed incidence of 1% is likely underestimated due to the limited use of echocardiographic evaluation, particularly TEE, which remains the gold standard for diagnosis [20,32]. In our institution, TEE is routinely recommended by infectious diseases consultants for all SAB cases in line with current guidelines; however, echocardiography is performed by cardiology, where the practice is to first perform TTE and proceed to TEE only if suspicious lesions are identified. This practice pattern, rather than unavailability of TEE, explains the very low rate observed in our cohort. In our cohort, cases of endocarditis were considered complications arising from central venous catheter-related bolldstream infections. The presence of an intravascular catheter facilitates the formation of a biofilm layer—a key virulence factor of *S. aureus*—which shields the bacteria from host phagocytic mechanisms. This biofilm barrier hampers treatment efficacy and is associated with increased mortality [33]. Central venous catheters act not only as portals of entry for microorganisms but also contribute to the persistence of infection by enabling microbial survival within biofilms. In our study, the presence of central venous catheter was identified as a significant risk factor for mortality, reinforcing existing literature that highlights the detrimental impact of foreign bodies on infection outcomes. This finding underscores the critical importance of effective catheter management strategies in clinical practice to reduce infection-related complications and mortality. In our cohort, mortality was 29% in patients whose catheter was removed, compared with 72% in those whose catheter was not removed and 23% in patients without a catheter. These findings highlight the critical role of catheter management, and suggest that failure to remove the central venous catheter may contribute to adverse outcomes in SAB.

This study has certain limitations. Firstly, the single-center design restricts the generalizability of the findings to broader populations. Additionally, as data collection was conducted retrospectively, patients with incomplete data were excluded, which constitutes another important limitation. TTE was performed in 60% of patients and TEEin only 1%; thus, it is likely that some cases of endocarditis were misclassified under the primary bacteremia group. This limitation may have led to an underestimation of endocarditis prevalence. Another limitation of our study is that we were unable to compare abbreviated scoring systems such as qPitt and qSOFA with their original versions, as not all required variables were consistently available in our dataset. Future multicenter, prospective studies should investigate whether these simplified tools perform comparably to the full PBS and SOFA scores in predicting outcomes of SAB.

## Conclusion

In conclusion, this single-center study, conducted in a high-risk cohort with a substantial proportion of patients with multiple comorbidities and immunosuppression, demonstrated that a high PBS (PBS ≥4) was the strongest prognostic indicator of mortality in SAB (OR: 11.72). Furthermore, the increase in odds ratio from 5.19 in univariate to 6.36 in multivariate analysis underscores that an elevated NLR (NLR >21) represents an independent prognostic factor, irrespective of other inflammatory markers. As our study population reflects a high-risk cohort, these findings provide valuable insights into outcomes in vulnerable patients; however, validation in more generalizable, multicenter cohorts is warranted to confirm their applicability.

## Supplementary Material

suppl_data.zip

## Data Availability

The study data information is available from the corresponding author on reasonable request.

## References

[1] Cheung GYC, Bae JS, Otto M. Pathogenicity and virulence of *Staphylococcus aureus*. Virulence. 2021;12(1):547–569. doi: 10.1080/21505594.2021.1878688. 33522395 PMC7872022

[2] Bai AD, Lo CKL, Komorowski AS, et al. *Staphylococcus aureus* bacteraemia mortality: a systematic review and meta-analysis. Clin Microbiol Infect. 2022;28(8):1076–1084. doi: 10.1016/j.cmi.2022.03.015. 35339678

[3] Chow JW, Yu VL. Combination antibiotic therapy versus monotherapy for gram-negative bacteraemia: a commentary. Int J Antimicrob Agents. 1999;11(1):7–12. doi: 10.1016/s0924-8579(98)00060-0. 10075272

[4] de Jager CP, van Wijk PT, Mathoera RB, et al. Lymphocytopenia and neutrophil-lymphocyte count ratio predict bacteremia better than conventional infection markers in an emergency care unit. Crit Care. 2010;14(5):R192. doi: 10.1186/cc9309. 21034463 PMC3219299

[5] Shen Y, Huang X, Zhang W. Platelet-to-lymphocyte ratio as a prognostic predictor of mortality for sepsis: interaction effect with disease severity—a retrospective study. BMJ Open. 2019;9(1):e022896. doi: 10.1136/bmjopen-2018-022896. PMC6352809 30782690

[6] Benfield T, Espersen F, Frimodt-Møller N, et al. Increasing incidence but decreasing in-hospital mortality of adult Staphylococcus aureus bacteraemia between 1981 and 2000. Clin Microbiol Infect. 2007;13(3):257–263. doi: 10.1111/j.1469-0691.2006.01589.x. 17391379

[7] Skov R, Gottschau A, Skinhøj P, et al. Staphylococcus aureus bacteremia: a 14-year nationwide study in hematological patients with malignant disease or agranulocytosis. Scand J Infect Dis. 1995;27(6):563–568. doi: 10.3109/00365549509047068. 8685634

[8] González-Barca E, Carratalà J, Mykietiuk A, et al. Predisposing factors and outcome of Staphylococcus aureus bacteremia in neutropenic patients with cancer. Eur J Clin Microbiol Infect Dis. 2001;20(2):117–119. doi: 10.1007/pl00011241. 11305464

[9] Horan TC, Andrus M, Dudeck MA. CDC/NHSN surveillance definition of health care–associated infection and criteria for specific types of infections in the acute care setting. Am J Infect Control. 2008;36(5):309–332. doi: 10.1016/j.ajic.2008.03.002. 18538699

[10] Paterson DL, Ko W-C, Von Gottberg A, et al. International prospective study of Klebsiella pneumoniae bacteremia: implications of extended-spectrum beta-lactamase production in nosocomial infections. Ann Intern Med. 2004;140(1):26–32. doi: 10.7326/0003-4819-140-1-200401060-00008. 14706969

[11] Vincent JL, Moreno R, Takala J, et al. The SOFA (Sepsis-related Organ Failure Assessment) score to describe organ dysfunction/failure. Intensive Care Med. 1996;22(7):707–710. doi: 10.1007/BF01709751. 8844239

[12] Friedman ND, Kaye KS, Stout JE, et al. Health care–associated bloodstream infections in adults: a reason to change the accepted definition of community-acquired infections. Clin Infect Dis. 2002;35(8):1061–1067. doi: 10.1086/344223. 12435215

[13] Minter DJ, Appa A, Chambers HF, et al. Contemporary management of Staphylococcus aureus bacteremia—controversies in clinical practice. Clin Infect Dis. 2023;77(11):e57–e68. doi: 10.1093/cid/ciad500. 37950887 PMC11959183

[14] van Hal SJ, Jensen SO, Vaska VL, et al. Predictors of mortality in *Staphylococcus aureus* bacteremia. Clin Microbiol Rev. 2012;25(2):362–386. doi: 10.1128/CMR.05022-11. 22491776 PMC3346297

[15] Allard C, Carignan A, Bergevin M, et al. Secular changes in incidence and mortality associated with *Staphylococcus aureus* bacteraemia in Quebec, Canada, 1991–2005. Clin Microbiol Infect. 2008;14(5):421–428. doi: 10.1111/j.1469-0691.2008.01965.x. 18325037

[16] Kıran P, Batirel A, Gençer S. Prediction of mortality in patients with sepsis due to gram-negative bacteremia: pitt Bacteremia Score, qSOFA, SIRS. FLORA İnfeksiyon Hastalıkları Dergisi. 2021;26(4):663–669. doi: 10.5578/flora.20219611.

[17] Salciccioli JD, Marshall DC, Pimentel MAF, et al. The association between the neutrophil-to-lymphocyte ratio and mortality in critical illness: an observational cohort study. Crit Care. 2015;19(1):13. doi: 10.1186/s13054-014-0731-6. 25598149 PMC4344736

[18] Huang Z, Fu Z, Huang W, et al. Prognostic value of neutrophil-to-lymphocyte ratio in sepsis: a meta-analysis. Am J Emerg Med. 2020;38(3):641–647. doi: 10.1016/j.ajem.2019.10.023. 31785981

[19] Romero-Vivas J, Rubio M, Fernandez C, et al. Mortality associated with nosocomial bacteremia due to methicillin-resistant *Staphylococcus aureus*. Clin Infect Dis. 1995;21(6):1417–1423. doi: 10.1093/clinids/21.6.1417. 8749626

[20] Fowler VG, Olsen MK, Corey GR, et al. Clinical identifiers of complicated *Staphylococcus aureus* bacteremia. Arch Intern Med. 2003;163(17):2066–2072. doi: 10.1001/archinte.163.17.2066. 14504120

[21] Palraj BR, Sohail MR. Appropriate use of echocardiography in managing *Staphylococcus aureus* bacteremia. Expert Rev anti Infect Ther. 2012;10(4):501–508. doi: 10.1586/eri.12.22. 22512758

[22] Willcox PA, Rayner BL, Whitelaw DA. Community-acquired *Staphylococcus aureus* bacteraemia in patients who do not abuse intravenous drugs. QJM. 1998;91(1):41–47. doi: 10.1093/qjmed/91.1.41. 9519211

[23] Solís N, Pérez C, Ramírez M, et al. Clinical presentation and microbiological characteristics of community-acquired *Staphylococcus aureus* bacteraemia at a tertiary hospital in Costa Rica. J Med Microbiol. 2024;73(9). doi: 10.1099/jmm.0.001883. 39234813

[24] Tong SYC, Fowler VG, Jr, Skalla L, et al. Management of *Staphylococcus aureus* bacteremia: a review. JAMA. 2025;334(9):798–808. doi: 10.1001/jama.2025.4288. 40193249 PMC12663922

[25] Mouton CP, Bazaldua OV, Pierce B, et al. Common infections in older adults. Am Fam Physician. 2001;63(2):257–268. 11201692

[26] Laupland KB, Ross T, Gregson DB. Staphylococcus aureus bloodstream infections: risk factors, outcomes, and the influence of methicillin resistance in Calgary, Canada, 2000–2006. J Infect Dis. 2008;198(3):336–343. doi: 10.1086/589717. 18522502

[27] Botheras CL, Bowe SJ, Cowan R, et al. C-reactive protein predicts complications in community-associated *Staphylococcus aureus* bacteraemia: a cohort study. BMC Infect Dis. 2021;21(1):312. doi: 10.1186/s12879-021-05962-7. 33794783 PMC8015062

[28] Kaasch AJ, Barlow G, Edgeworth JD, et al. *Staphylococcus aureus* bloodstream infection: a pooled analysis of five prospective, observational studies. J Infect. 2014;68(3):242–251. doi: 10.1016/j.jinf.2013.10.015. 24247070 PMC4136490

[29] Fowler VG, Li J, Corey GR, et al. Role of echocardiography in evaluation of patients with *Staphylococcus aureus* bacteremia: experience in 103 patients. J Am Coll Cardiol. 1997;30(4):1072–1078. doi: 10.1016/s0735-1097(97)00250-7. 9316542

[30] Kaasch AJ, Fowler VG, Rieg S, et al. Use of a simple criteria set for guiding echocardiography in nosocomial *Staphylococcus aureus* bacteremia. Clin Infect Dis. 2011;53(1):1–9. doi: 10.1093/cid/cir320. 21653295 PMC3149212

[31] Lau L, Wiens EJ, Karlowsky JA, et al. Clinical utility of echocardiography for the diagnosis of native valve infective endocarditis in Staphylococcus aureus bacteremia. Echocardiography. 2019;36(10):1852–1858. doi: 10.1111/echo.14480. 31536152

[32] Murdoch DR, Corey GR, Hoen B, et al. Clinical presentation, etiology, and outcome of infective endocarditis in the 21st century: the International Collaboration on Endocarditis–Prospective Cohort Study. Arch Intern Med. 2009;169(5):463–473. doi: 10.1001/archinternmed.2008.603. 19273776 PMC3625651

[33] Guo H, Tong Y, Cheng J, et al. Biofilm and small colony variants—an update on Staphylococcus aureus strategies toward drug resistance. Int J Mol Sci. 2022;23(3):1241. doi: 10.3390/ijms23031241. 35163165 PMC8835882

