# Supplementary material for: Clinical and laboratory predictors of mortality in Staphylococcus aureus bacteremia in a high-risk setting: a single-center retrospective analysis of Pitt score, SOFA, neutrophil-to-lymphocyte ratio, and platelet-to-lymphocyte ratio
Source: Ann Med. 2025 Oct 21;57(1):2573984. doi: 10.1080/07853890.2025.2573984 (PMC12541918; doi:10.1080/07853890.2025.2573984)
Supplement: suppl_data.zip [file IANN_A_2573984_SM3742.zip › suppl_data/Suplementary table 1.docx]

**Supplementary Table 1.** Distribution of comorbid conditions among patients with Staphylococcus aureus bacteraemia

| **Comorbidity** | **n** | **%** |
| --- | --- | --- |
| Hypertension | 74 | 49 |
| Diabetes mellitus | 58 | 38.4 |
| Cardiovascular disease | 44 | 29.1 |
| Chronic kidney disease | 41 | 27.1 |
| Malignancy | 32 | 21.2 |
| Chronic obstructive pulmonary disease | 17 | 11.3 |
| Stroke | 10 | 6.6 |
| Alzheimer’s disease | 11 | 7.3 |
| Rheumatologic disease | 8 | 5.3 |
| Renal transplantation | 3 | 2.0 |
| Other | 20 | 13.3 |
