# Supplementary material for: Clinical and laboratory predictors of mortality in Staphylococcus aureus bacteremia in a high-risk setting: a single-center retrospective analysis of Pitt score, SOFA, neutrophil-to-lymphocyte ratio, and platelet-to-lymphocyte ratio
Source: Ann Med. 2025 Oct 21;57(1):2573984. doi: 10.1080/07853890.2025.2573984 (PMC12541918; doi:10.1080/07853890.2025.2573984)
Supplement: suppl_data.zip [file IANN_A_2573984_SM3742.zip › suppl_data/Suplementary table 2.docx]

**Supplementary Table 2.** Distribution of immunosuppressive conditions among patients with Staphylococcus aureus bacteraemia

| **Immunosuppressive conditions** | **n** | **%** |
| --- | --- | --- |
| Malignancy | 32 | 41.05 |
| Burn injury | 32 | 41.05 |
| Corticosteroid use | 11 | 14.1 |
| Renal transplantation | 3 | 3.8 |
