# Supplementary material for: Clinical and laboratory predictors of mortality in Staphylococcus aureus bacteremia in a high-risk setting: a single-center retrospective analysis of Pitt score, SOFA, neutrophil-to-lymphocyte ratio, and platelet-to-lymphocyte ratio
Source: Ann Med. 2025 Oct 21;57(1):2573984. doi: 10.1080/07853890.2025.2573984 (PMC12541918; doi:10.1080/07853890.2025.2573984)
Supplement: suppl_data.zip [file IANN_A_2573984_SM3742.zip › suppl_data/Suplementary table 3.docx]

**Supplementary Table 3.** Distribution of Staphylococcus aureus bacteraemia according to Friedman classification

| **Classification** | **n** | **%** |
| --- | --- | --- |
| Healthcare-associated (HCA-SAB) | 86 | 57.3 |
| Hospital-acquired (HA-SAB) | 38 | 25.3 |
| Community-acquired (CA-SAB) | 26 | 17.3 |
| Total | 150 | 100 |
