# Supplementary material for: Clinical and laboratory predictors of mortality in Staphylococcus aureus bacteremia in a high-risk setting: a single-center retrospective analysis of Pitt score, SOFA, neutrophil-to-lymphocyte ratio, and platelet-to-lymphocyte ratio
Source: Ann Med. 2025 Oct 21;57(1):2573984. doi: 10.1080/07853890.2025.2573984 (PMC12541918; doi:10.1080/07853890.2025.2573984)
Supplement: suppl_data.zip [file IANN_A_2573984_SM3742.zip › suppl_data/Supplementary Table 4.docx]

**Supplementary Table 4**. Comparison of demographic and clinical characteristics between MRSA and MSSA bacteremia

| Variable | | Overall  (n=150) | | MRSA  (n=45) | | MSSA  (n=105) | | P |
| --- | --- | --- | --- | --- | --- | --- | --- | --- |
| Age | >65 | 76 | 51% | 21 | 47% | 55 | 52% | 0.643 |
|  | ≤65 | 74 | 49% | 24 | 53% | 50 | 48% |  |
| Sex | Male | 83 | 55% | 23 | 51% | 60 | 57% | 0.616 |
|  | Female | 67 | 45% | 22 | 49% | 45 | 43% |  |
| Comorbidity | Yes | 132 | 88% | 34 | 76% | 98 | 93% | **0.005** |
|  | No | 18 | 12% | 11 | 24% | 7 | 7% |  |
| Immunosuppression | Yes | 78 | 52% | 24 | 53% | 54 | 51% | 0.972 |
|  | No | 72 | 48% | 21 | 47% | 51 | 49% |  |
| Hospitalization within the past 3 months | Yes | 86 | 57% | 26 | 58% | 60 | 57% | 1.000 |
|  | No | 64 | 43% | 19 | 42% | 45 | 43% |  |
| Hospitalization within the past 12 months | Yes | 99 | 66% | 29 | 64% | 70 | 67% | 0.940 |
|  | No | 51 | 34% | 16 | 36% | 35 | 33% |  |
| Clinical presentation of infection | Primary bacteremia | 48 | 32% | 15 | 33% | 33 | 31% | na |
|  | Pneumonia | 46 | 31% | 16 | 36% | 30 | 29% |  |
|  | Skin and soft tissue infection | 23 | 15% | 4 | 9% | 19 | 18% |  |
|  | Urinary tract infection | 17 | 11% | 8 | 18% | 9 | 9% |  |
|  | Catheter infection | 11 | 7% | 2 | 4% | 9 | 9% |  |
|  | Trombophlebitis | 3 | 2% | 0 | 0% | 3 | 3% |  |
|  | Endocarditis | 2 | 1% | 0 | 0% | 2 | 2% |  |
| Epidemiologic classification of infection | Community-acquired | 26 | 17.3% | 8 | 30.8% | 18 | 69.2% | 1.000 |
|  | Healthcare-associated | 86 | 57.3% | 26 | 30.2% | 60 | 69.8% |  |
|  | Hospital-acquired | 38 | 25.3% | 11 | 28.9% | 27 | 71.1% |  |
| Catheter Presence | Yes | 112 | 75% | 34 | 76% | 78 | 74% | 1.000 |
|  | No | 38 | 25% | 11 | 24% | 27 | 26% |  |
| Pitt Score | >4 | 50 | 33% | 13 | 29% | 37 | 35% | 0.571 |
|  | ≤4 | 100 | 67% | 32 | 71% | 68 | 65% |  |
| SOFA Score | ≥2 | 124 | 83% | 37 | 82% | 87 | 83% | 1.000 |
|  | <2 | 26 | 17% | 8 | 18% | 18 | 17% |  |
| Mortality | Non-survivors | 69 | 46% | 25 | 56% | 44 | 42% | 0.174 |
|  | Survivors | 81 | 54% | 20 | 44% | 61 | 58% |  |

*P* values were calculated using the Mann–Whitney U test.
