# Supplementary material for: Clinical and laboratory predictors of mortality in Staphylococcus aureus bacteremia in a high-risk setting: a single-center retrospective analysis of Pitt score, SOFA, neutrophil-to-lymphocyte ratio, and platelet-to-lymphocyte ratio
Source: Ann Med. 2025 Oct 21;57(1):2573984. doi: 10.1080/07853890.2025.2573984 (PMC12541918; doi:10.1080/07853890.2025.2573984)
Supplement: suppl_data.zip [file IANN_A_2573984_SM3742.zip › suppl_data/Supplementary Table 5.docx]

**Supplementary Table 5.** Comparison of neutrophil and lymphocyte counts according to fever status

| **Variable** | **Fever present (N=75) Median (Q1–Q3)** | **Fever absent (N=75) Median (Q1–Q3)** | **p-value** |
| --- | --- | --- | --- |
| Neutrophils (/mm³) | 9370 (6430–13120) | 12010 (6190–17330) | 0.065 |
| Lymphocytes (/mm³) | 870 (590–1240) | 950 (430–1340) | 0.886 |
